# Postural adjustment as a function of scene orientation

**DOI:** 10.1167/jov.22.4.1

**Published:** 2022-03-02

**Authors:** Kanon Fujimoto, Hiroshi Ashida

**Affiliations:** 1 Department of Psychology, Graduate School of Letters, Kyoto University, Kyoto, Japan; 2 Japan Society for the Promotion of Science, Tokyo, Japan; 3 Department of Psychology, Graduate School of Letters, Kyoto University, Kyoto, Japan

**Keywords:** postural control, orientation, gravity, coordinate, perception and action

## Abstract

Visual orientation plays an important role in postural control, but the specific characteristics of postural response to orientation remain unknown. In this study, we investigated the relationship between postural response and the subjective visual vertical (SVV) as a function of scene orientation. We presented a virtual room including everyday objects through a head-mounted display and measured head tilt around the naso-occipital axis. The room orientation varied from 165° counterclockwise to 180° clockwise around the center of display in 15° increments. In a separate session, we also conducted a rod adjustment task to record the participant's SVV in the tilted room. We applied a weighted vector sum model to head tilt and SVV error and obtained the weight of three visual cues to orientation: frame, horizon, and polarity. We found significant contributions for all visual cues to head tilt and SVV error. For SVV error, frame cues made the largest contribution, whereas polarity contribution made the smallest. For head tilt, there was no clear difference across visual cue types, although the order of contribution was similar to the SVV. These findings suggest that multiple visual cues to orientation are involved in postural control and imply different representations of vertical orientation across postural control and perception.

## Introduction

Maintaining an upright stance is the basic function of the human postural control system. To achieve this, humans use multisensory information from vestibular, proprioceptive, and visual inputs to estimate body position, orientation, and movement from environmental coordinates. Vision especially plays an important role in fine postural control. For example, the magnitude of postural sway increases as visual acuity decreases (Paulus, Straube, & Brandt, 1984), and is doubled when the eyes are closed compared to when they are open (Edwards, 1946). Moreover, the postural system depends largely on vision in unfamiliar postural situations, such as standing on one foot (Lee & Lishman, 1977).

Visual control of posture may utilize two types of visual information. One is dynamic visual motion, which contains information about body movement, and the other is static visual orientation, which contains information about body tilt relative to gravity direction. The former, known as optic flow, induces postural sways of a standing observer that counteract perceived self-motion (a visually evoked postural response) (Bronstein, 1986). Although this type of postural control has been well documented and modeled (e.g., Palmisano, Allison, Schira, & Barry, 2015; Wei, Stevenson, & Körding, 2010), few studies have investigated the latter static effects. Stabilization of balance is essential for standing, and the postural system helps to achieve this by aligning the body axis with gravity. Indeed, some studies have reported that a tilted visual scene can induce postural inclination toward the scene tilt (Guerraz, Yardley, Bertholon, Pollak, Rudge, Gresty, & Bronsteinn, 2001; Isableu, Ohlmann, Crémieux, & Amblard, 1997; Ohmura, Yano, Katsuhira, Migita, Yozu, & Kondo, 2017; Tsuruhara & Kaneko, 2006). However, exactly how visual tilt influences postural control is not sufficiently understood.

The visual–perceptual system might share the same spatial representation coordinates with postural control. Perception of verticality has often been studied by asking participants to adjust the orientation of a visual rod to their perceived direction of gravity. The orientation of the adjusted rod is called the observer's subjective visual vertical (SVV). The SVV is often affected by visual information about orientation. For example, a tilted square frame often induces an illusory tilt of the vertical rod in the opposite orientation of the frame tilt (rod-and-frame illusion), and, accordingly, the SVV shifts in the direction of the frame tilt (Witkin & Asch, 1948). The rod-and-frame illusion also induces the illusory sensation of the observer's body tilting, which can be measured by the manual adjustment of a chair or the head to the subjective vertical (Cian, Esquivié, Barraud, & Raphel, 1995; Ebenholtz & Benzschawel, 1977; Sigman, Goodenough, & Flannagan, 1979). The perceptual illusion could be induced as a secondary effect of the shift of environmental coordinates to the frame orientation (Dassonville & Reed, 2015; Ebenholtz & Benzschawel, 1977; Sigman et al., 1979), and the coordinates might be shared by the postural system, producing a postural response to the frame orientation.

The degree of SVV error from the gravitational vertical does not linearly increase with frame orientation, but rather shows a sinusoidal modulation with a 90° period to the frame orientation (Alberts, de Brouwer, Selen, & Medendorp, 2016; Beh, Wenderoth, & Purcell, 1971; Oltman, 1968; Vingerhoets, De Vrijer, Van Gisbergen, & Medendorp, 2009). This is plausible because a square frame itself has a 90° period when rotated. On the other hand, the rod-and-frame effect can be produced even by a single peripheral line (Li & Matin, 2005a, 2005b), and the effect still has a 90° period (Vingerhoets et al., 2009). Independent manipulation of each single line of a square frame reveals an additive effect of lines rather than the effect of frame configuration (Lunsford & Dannemiller, 2006). In this context, the cyclical modulation of the SVV might be produced because of the ambiguity of lines as cues to the vertical direction; the line might be along the gravitational vertical or perpendicular to it. In Mittelstaedt's model (Mittelstaedt, 1983, 1986), such cyclical modulation of the SVV could be generated by the sum of weighted sensory vectors representing gravity (detected by the vestibular organ), body axis (idiotropic vector), visual orientation, and vector lengths representing their relative weights (for an intuitive explanation of the model, see figure 5 in Dyde, Jenkin, & Harris, 2006). Although the sinusoidal modulation of SVV has been well documented and modeled, it is still unclear whether the postural response to a tilted scene has a similar angular function of visual orientation.

Visual scenes have multiple components of visual cues to orientation (Haji-Khamneh & Harris, 2009; Harris, Dyde, & Jenkin, 2007; Harris, Jenkin, Dyde, & Jenkin, 2011; Mittelstaedt, 1986). Mittelstaedt (1986) decomposed the visual cues for orientation into one-, two-, and four-fold components. Harris et al. (2007) referred to these three visual components as *frame*, *horizon*, and *polarity* cues. The frame cue (fourfold, 90° period) is similar to the square frame in the rod-and-frame illusion and has four possible directions of “up.” It is also derived from lines in the overall scene. A horizon cue (twofold, 180° period) is a line specifying the elevation of the horizon even when it is not directly visible (Haji-Khamneh & Harris, 2009). Polarity cues (onefold, 360° period) are derived from daily objects, such as furniture or vehicles, which provide absolute direction of upward based on prior knowledge that the most familiar orientation of the object would align with gravity. Polarity cues are also derived from the spatial layout of natural features such as the clouds are always in the sky, and the sky is always above the mountains. Harris et al. (2007) and Haji-Khamneh and Harris (2009) examined the perceptual upright (PU) using a letter-recognition task and explained their results using the vector sum model of these three components. Although the PU is closely related to the SVV, their PU estimates involved higher object recognition processes, whereas the SVV focuses on verticality more directly. Although those visual cues could determine the SVV (Mittelstaedt, 1986), whether and to what extent those orientation cues substantially contribute to postural adjustment is unclear.

In this study, we investigated postural response and SVV when viewing an everyday scene to identify the characteristics of postural response when compared with the perceived visual orientation of the environment. We presented a tilted virtual room through a head-mounted display (HMD) and measured head tilt using a tracking system in the HMD while participants were standing. To focus on the effects of static tilt, we presented the room without dynamic rotation, in a random order and with intervals of darkness between trials. In a separate session, we also conducted a rod adjustment task to measure the participants’ SVV in the same HMD-presented tilted room, where participants adjusted a visual rod in the room by a gamepad while sitting on a stool. We applied the vector sum model of Mittelstaedt (1986) to both head tilt and SVV results and estimated the weights of the three visual components (frame, horizon, and polarity) by fitting a function with three sinusoidal components. We then tested whether each visual component substantially contributed to the results and compared contributions to the head tilt and SVV. We also tested the correlation between the head tilt and SVV, based on the hypothesis that perceived body tilt induced by scene tilt (Cian et al., 1995; Ebenholtz & Benzschawel, 1977; Sigman et al., 1979) would produce a compensatory postural response. Although postural sway is often measured by the center of foot pressure (CoP), we measured head tilt to compare postural response with SVV orientation. Head tilt also represents overall body inclination, as head movement often follows the CoP (Guerraz & Bronstein, 2008; Tanahashi, Ujike, Kozawa, & Ukai, 2007), whereas other body segments also compensate for scene orientation.

## Methods

### Participants

Sixteen participants (undergraduate and graduate students from Kyoto University) took part in the main experiment (eight males and eight females; mean age = 21.88 years, *SD* = 2.03). All participants had normal or corrected-to-normal visual acuity with contact lenses. None had a history of vestibular disorders. They were informed about the purpose of the study, and they gave written consent for the procedure, which was conducted in accordance with the tenets of the Declaration of Helsinki and approved by the local ethics committee of Kyoto University (2-P-3).

### Apparatus and stimuli

Visual stimuli were presented on an Oculus Rift CV1 HMD (Meta Platforms, Inc., Menlo Park, CA), which has a pair of organic light-emitting diode displays with a display resolution of 1080 × 1200 pixels/eye and a refresh rate of 90 Hz. The field of view of the display was approximately 110° diagonally. The HMD position and orientation were recorded at a rate of 50 Hz by using the tracking system of the Oculus Rift. The experiment was conducted with a Windows 10 PC (Microsoft, Redmond, WA). A Microsoft Xbox One gamepad was used to record participants’ responses.

**Figure 1.**
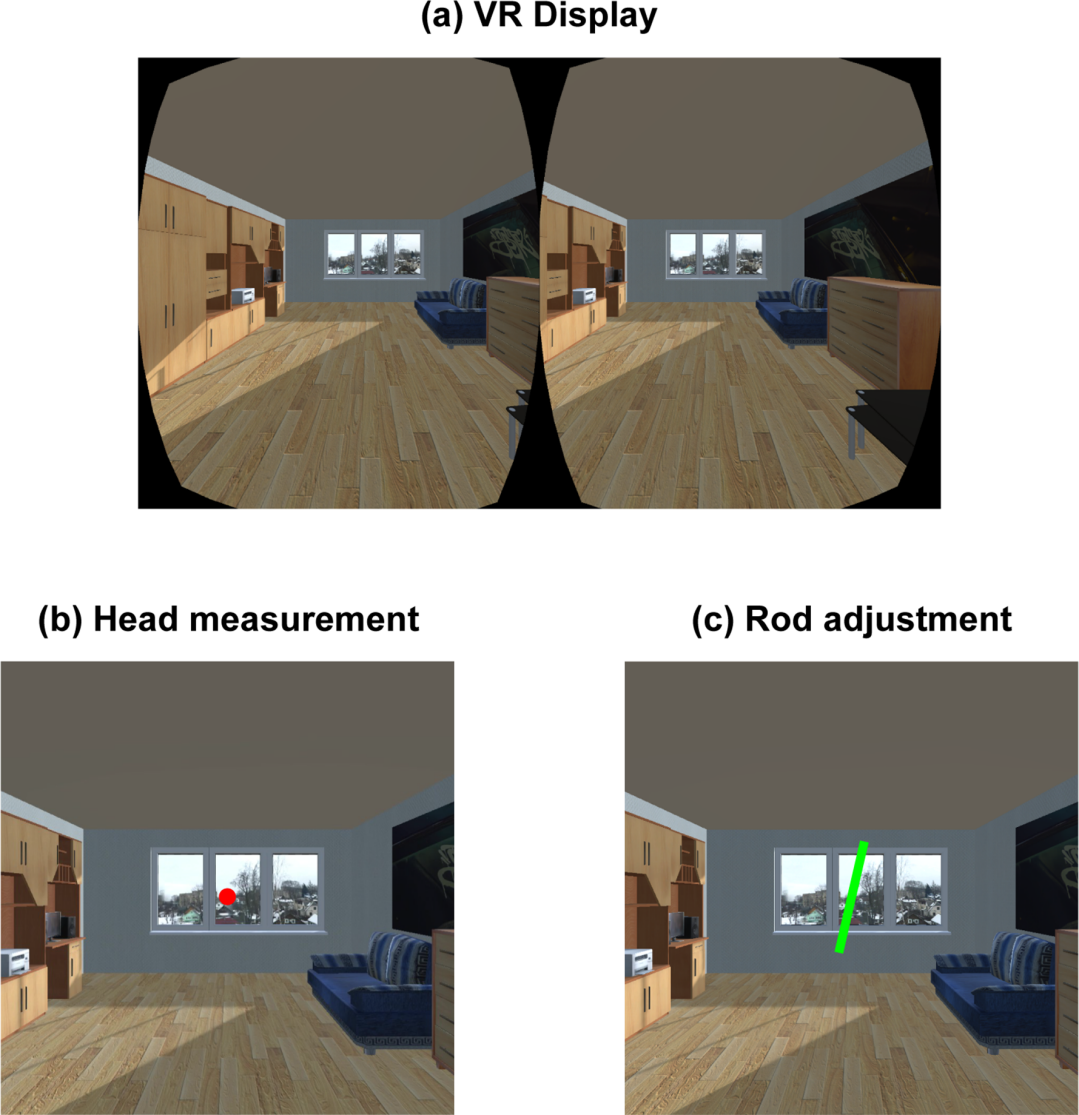
(a) A virtual room as viewed through a head-mounted display. In the head measurement task (b), participants counted the frequency of red dot flashes. In the rod adjustment task (c), participants adjusted the orientation of the visual rod to the gravitationally vertical. Here, the dot and the rod have been enlarged for clarity.

Virtual reality scenes were created with the three-dimensional Unity 5.3.1 (Unity Technologies, San Francisco, CA). A furnished virtual room (Figure 1) was presented on the HMD with binocular disparity. The simulated room was 17.5 m in width, 7 m in height, and 25 m in depth. The virtual cameras that simulated participants’ binocular viewpoints were positioned at a height of 4 m, just in front of the back wall, equidistant from the sidewalls, and orientated straight ahead toward the front wall and windows. The simulated height of the camera was set much higher than usual body height because of the common underestimation of distance in virtual reality environments (Renner, Velichkovsky, & Helmert, 2013). The room was tilted along the line of sight at one of 24 roll orientations from −165° to 180° in 15° increments. The layout of the room was mirrored in half of the trials. To measure head movement, a fixation dot was positioned at the center of the display, which changed color at 3 Hz. The fixation color varied among red, green, blue, and yellow. In the rod adjustment task, it was replaced with a green rod, extending 17° in length, 0.6° in width.

### Procedure

In the head movement measurement task, participants were instructed to stand still with their feet together throughout the stimulus observation. They held the gamepad in both hands in front of their body at waist height. In each trial, participants observed the virtual room presented for 10 s, during which roll orientation was presented in one of the 24 orientations. To fix their attention in the center of the display, they counted the number of times the red fixation dot turned on the screen via the gamepad. Their head movements were recorded during the observation. Every trial was followed by a short break for 10 s to reduce potential after-effects. During the break, a black background with the word “break” was displayed in white text in the center of the screen. After the break, two white disks (3.3° and 1.8° diameter) appeared to recalibrate the head position. The larger disk was located in the center of the room, and the smaller disk was located in the center of the HMD, which followed the participant's head movement. The participants hovered the smaller disk over the larger one by moving their heads to adjust their viewpoint straight ahead. If the participant maintained the angular distance of the center of the two disks at less than 1° for 3 s, the disks disappeared and the next trial started automatically. The head measurement consisted of eight blocks of 12 trials. Each block included 12 trials out of 24 room orientations. Each orientation was repeated four times. The room orientation order was randomized within each block. After each block was completed, participants took a break for as long as they needed.

In the rod adjustment task, participants were seated on a stool with their head unrestrained. They held the gamepad on their lap with both hands. Initial rod orientation was randomly assigned in each trial to avoid any influence of initial rod orientation (Hoppenbrouwers, Wuyts, & Van de Heyning, 2004). In each trial, they were asked to change the rod orientation in the virtual tilted room to appear gravitationally vertical using the directional pad on the gamepad. The rod orientation changed by ±2°/s when the left or right button was held down. If the button was pressed for more than 20 ms, the speed of the rod adjustment increased to 30°/s. When participants had made the appropriate adjustment, they pressed another button to finish the trial, and the next trial started immediately. The rod adjustment task consisted of four blocks of 24 trials, and each block included all 24 room orientations. Each orientation was repeated four times. The room orientation order was randomized within each block. After each block, participants took a break for as long as they needed.

The experiment was conducted over 2 days. On both days, the head measurement and rod adjustment tasks were completed. The order of the head measurement and rod adjustment tasks was counterbalanced across participants. Four blocks of the head measurement task and two blocks of the rod adjustment task were completed on both days. Before the first block of the head measurement and rod adjustment tasks on each day, participants completed several practice trials until they were familiar with the task.

### Data processing

For head tilt, we calculated the mean roll angle of the HMD during the stimulus presentation relative to the initial roll angle of the presentation on each trial. This mean head tilt was used as an index of postural response to the room tilt. In the rod adjustment task, final rod adjustments were collected to obtain the SVV errors relative to the gravitational vertical (0°) for each trial.

### Modeling

We estimated the effect of visual cues on head tilt and SVV error using the weighted vector sum model (Dyde et al., 2006; Mittelstaedt, 1983, 1986). This model assumes that the subjective vertical (SV) is composed of the sum of multiple vectors including gravity, body orientation and visual cues, represented by the formula,

(1) s=g+b+v

where ***s***, ***g***, ***b***, and ***v*** represent spatial vectors of SV, gravity, body axis, and visual orientation coordinates, respectively. In this study, we assessed the SVV error only for the frontal plane. Thus, we treated them as planar vectors with horizontal and vertical components and ignored the depth components. The vector lengths represent their relative weights of contribution.

For convenience, we defined the tilt angle in a clockwise fashion, with upright being zero. As participants worked on the tasks in an upright posture only, the gravity and body vectors were always aligned in an upright orientation. Therefore, we treated these vectors as a single unit vector:

(2) g+b=01

where the first and second components on the right side represent the horizontal and vertical components, respectively. The length of the gravity-body vector was defined as 1, and the visual vector was expressed relative to the gravity-body vector.

We broke down the visual vector into three visual components with different periods: frame (90°), horizon (180°), and polarity (360°) (Haji-Khamneh & Harris, 2009). Thus, ***v*** is expanded as follows:

(3) v=vf+vh+vp=wfcos4θsin4θ+whcos2θsin2θ+wpcosθsinθ

where ***v_f_***, ***v_h_***, and ***v_p_*** represent the vectors of frame, horizon, and polarity cues, respectively. Each vector consists of cosine and sine functions of different periods and the weight (length) of *w_f_*, *w_h_*, or *w_p_*. θ is the room orientation in degrees. These equations are summarized as

(4) sshsv=wf·cos4θ+wh·cos2θ+wp·cosθ1+wf·sin4θ+wh·sin2·θ+wp·sinθ

The two vector elements, *s_h_* and *s_v_*, are the horizontal and vertical components of the SV. Then we took the inverse tangent of ***s*** to obtain the angle of orientation. We also introduced an offset angle (α) for possible individual bias of the SV. Thus, the current model has four free parameters, including the three visual weights and the overall bias, expressed by the following formula:

(5) $$\varphi =\alpha +\tan^{-1}\frac{s_{h}}{s_{v}}$$

Importantly, the model assumes that the observer would adjust their head or rod orientation to ***s***. Thus, φ represents the amount of head tilt or SVV error.

We fitted the model to our data using nonlinear mixed-effect model data analyses and the *nlmer* function in the R package *lme4* (Bates, Mächler, Bolker, & Walker, 2015). We used R 4.0.3 (R Core Team, 2020). Only intercepts were treated as random effects by participants. Finally, four parameters were obtained for the head tilt and SVV error. In addition, we fitted the same model using the *nls* function and obtained the corresponding parameters for each participant to examine whether the magnitudes of the visual components for the head tilt and SVV error are correlated with each other across participants.

## Results

Out of 16 participants in the study, two finished the first day only. Thus, we collected data for only two trials per condition for these two participants. For the other participants, we collected data for four trials per condition.

**Figure 2.**
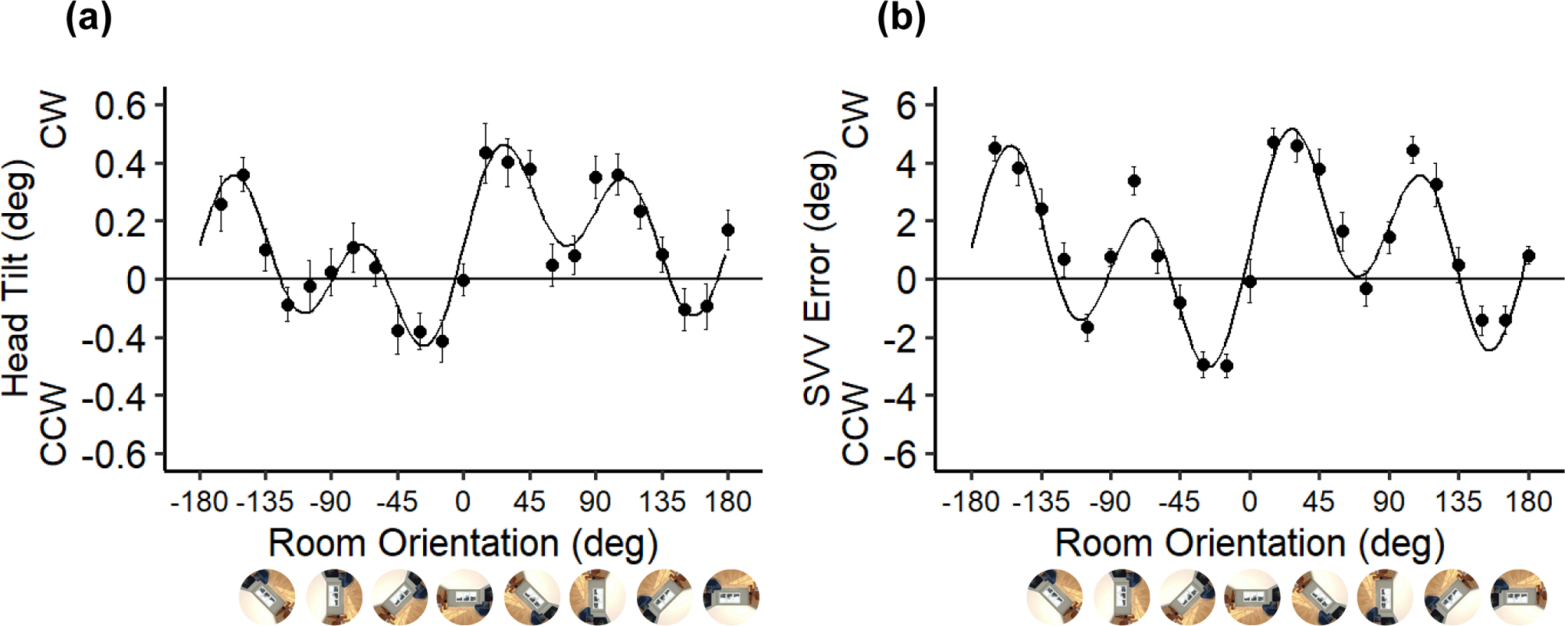
Mean head tilt biases (a) and SVV errors (b) as a function of room orientation. The positive and negative values of the SVV error indicate that the participants’ mean SVV shifted clockwise (CW) or counterclockwise (CCW) relative to the gravitational vertical, respectively. Error bars show standard errors of the mean across participants. Curves show the best fits of the vector sum model.

In Figure 2, the dot plots in the left and right panels show the mean head tilt and SVV error across participants during stimulus presentation as a function of room orientation. The black curves represent the best fit to the data. The best-fit parameter values are listed in Table 1. The weights of the three visual vectors were 10 times larger for the SVV error than for head tilt. The weight of the frame components tended to be the largest, followed by horizon components, and the polarity cues tended to be the smallest for both the head tilt and SVV. To examine to what extent each visual cue contributed to the head tilt and SVV error, we tested for the fitted parameters using *ggcoefstats* in the package *ggstatsplot*. The tests revealed that all visual components of *w_f_*, *w_h_*, and *w_p_* significantly influenced both the head tilt and SVV error, whereas the overall bias (α) values were not significant (Table 1).

**Table 1.** Best-fit parameters and their significance.

	Estimate	*t* (*df*)	*P*
Head tilt			
*w_f_*	3.50E-03	10.25 (1434)	<0.001
*w_h_*	2.18E-03	6.40 (1434)	<0.001
*w_p_*	2.14E-03	6.27 (1434)	<0.001
α	2.01E-03	1.85 (1434)	0.064
SVV error			
*w_f_*	4.77E-02	20.50 (1434)	<0.001
*w_h_*	2.63E-02	11.25 (1434)	<0.001
*w_p_*	1.36E-02	5.83 (1434)	<0.001
α	1.87E-02	1.76 (1434)	0.078

Using individual visual weights of head tilt and SVV error obtained from each participant, we conducted a within-subject ANOVA with two factors of the visual cue (frame, horizon, polarity) and task type (head tilt and SVV error). There were significant main effects of visual cue, *F*(2,30) = 25.57, *p* < 0.001, η^2^ = 0.115, and task type, *F*(1,15) = 44.05, *p* < 0.001, η^2^ = 0.382. There was also a significant interaction between visual cue and task type, *F*(2,30) = 20.27, *p* < 0.001, η^2^ = 0.098. Subsequent analysis revealed a significant simple main effect of visual cue for SVV error, *F*(2,30) = 23.05, *p* < 0.001, η^2^ = 0.346, and for head tilt, *F*(2,30) = 3.51, *p* = 0.043, η^2^ = 0.095. We also found simple main effects of task type for all of the visual cues: frame, *F*(1,15) = 80.15, *p* < 0.001, η^2^ = 0.725; horizon, *F*(1,15) = 16.23, *p* = 0.001, η^2^ = 0.358; and polarity, *F*(1,15) = 8.84, *p* = 0.001, η^2^ = 0.221. We then carried out multiple comparisons of the visual cues for head tilt but found only marginal differences between frame and horizon cues (*t*_15_ = 2.63, *p* = 0.057, *d* = 0.605), and frame and polarity cues (*t*_15_ = 2.46, *p* = 0.057, *d* = 0.739). There was no significant difference between horizon and polarity cues (*t*_15_ = 0.04, *p* = 0.965, *d* = 0.015). Multiple comparisons of the visual cues for SVV error revealed that the largest contribution came from frame cues compared with horizon cues (*t*_15_ = 3.50, *p* = 0.003, *d* = 0.977) and polarity cues (*t*_15_ = 7.50, *p* < 0.001, *d* = 1.880). The smallest contribution came from polarity cues compared with frame cues and horizon cues (*t*_15_ = 2.89, *p* = 0.011, *d* = 0.591). Familywise errors were corrected with Shaffer's modified sequentially rejective Bonferroni procedure, and the adjusted *p* values were used for the multiple comparisons. In summary, the weights of the visual cues were much larger for the SVV error than head tilt. For the SVV error, the frame contribution was the largest, followed by the horizon contribution and then the polarity contribution. For head tilt, there was no clear difference across visual cue types, although the order of contribution was similar to the SVV.

To test for a possible relationship between postural response and SVV error, we carried out correlational analyses across participants between the corresponding visual cues of the head tilt and SVV error, using the individual best-fit parameters obtained for each participant (Table 2). However, there was no significant correlation with any of the three cues.

**Table 2.** Correlations of individual parameters between the head tilt and SVV error.

Parameter	*r*	*P*
*w_f_*	0.075	0.784
*w_h_*	−0.157	0.561
*w_p_*	0.208	0.440

## Discussion

### Summary of results

We examined postural response and the SVV to the visual orientation of a virtual room viewed through a head-mounted display. We found substantial contributions of all three types of visual cues (frame, horizon, and polarity) to both head tilt and SVV error. The frame cue made the largest contribution and the polarity cue made the smallest to the SVV error. The pattern was similar for head tilt, but the differences were not statistically significant. The visual cue contributions did not show significant correlations between SVV error and head tilt across participants.

### Substantial contribution of visual components

The contribution of frame cue is in line with research on the rod-and-frame effect (Alberts et al., 2016; Beh et al., 1971; Oltman, 1968; Vingerhoets et al., 2009) and its postural influence (Guerraz et al., 2001; Isableu et al., 1997; Ohmura et al., 2017; Tsuruhara & Kaneko, 2006). Previous studies on the effect of visual tilt on postural control only tested for specific orientations of the visual scene—in particular, the most effective orientation (around 20°) for the rod-and-frame effect. The current study examined the full angular function of the postural response to visual orientation and found a periodic change in head tilt, as well as the SVV error. The influence of the frame cues may have an ecological basis. Our visual environments, especially modern urban scenes, contain a lot of horizontal and vertical orientations (Coppola, Purves, McCoy, & Purves, 1998; Switkes, Mayer, & Sloan, 1978; Van Der Schaaf & Van Hateren, 1996). Based on prior knowledge about such scenes, the visual system can infer that frame lines in the scene would represent the direction of gravity or orientation perpendicular to gravity, biasing the conscious SVV or automatic postural stabilization to the line orientation. It should be noted that, although frame cue makes the largest contribution to SVV (and possibly head tilt) among the three types of visual orientation, its influence on head tilt was quite small. Because frame cues are ambiguous in terms of four possible upward directions, they might not be so reliable from an ecological perspective.

We found a moderate contribution of the horizon cue for both head tilt and SVV error, although there was no explicit horizon in the scene. One possible explanation for the horizon effect is the aspect ratio of the room. Harris, Jenkin, Jenkin, Dyde, and Oman (2010) reported that when participants chose a surface to stand on among the surrounding walls of a tilted room, the choice probability of each surface depended on the retinal area of each wall. As the room in the present study was rectangular, the floor and ceiling occupied larger retinal areas than the walls, and so might have been more relevant as the supporting surface than the walls, thus serving as implicit horizon cues. Although frame cues are based on line orientations, horizon cues might be supported by surface perception. However, further investigation is required to reveal the factors crucial for the horizon effect, as previous research considered the rod-and-frame effect as the summation of line effects, rather than being caused by frame configuration (Li & Matin, 2005a, 2005b; Lunsford & Dannemiller, 2006). The effect of aspect ratio might also be worth investigating.

The contribution of polarity cues implies involvement of object recognition processes in verticality perception and postural control (Haji-Khamneh & Harris, 2009), although their effects are relatively small. The cognitive effect on the SVV is consistent with a report that showed the effect of cognitive cues to orientation on the SVV (Cian, Raphel, & Barraud, 2001), such that a round clock with tilted dials induced a substantial rod-and-frame effect. Although participants in the study by Cian et al. (2001) might have only adjusted a rod to the preferred upright of the object, current results suggest more automatic cognitive processes as participants involuntarily adjusted their postures based on object polarity. In addition to object recognition, the overall layout of the scene might contribute to the polarity effect. As compared with detecting every objects in a scene, grasping the gist of the scene requires only a single glance (Friedman, 1979; Oliva, 2005; Oliva & Torralba, 2006). If polarity cues are derived from individual object recognition and scene layout, polarity effects might have two processing stages: one performed rapidly from getting the gist of the scene, and the other performed more slowly from individual object recognition. Future research should investigate the relative contributions of object and scene recognitions and examine the temporal characteristics of polarity effects.

### Relative contributions of visual components

Of the three types of visual cues, the relative contribution of the frame cues was the largest and that of the polarity cues was the smallest (which was significant at least for the SVV error). The current result conflicts somewhat with studies by Harris et al. (2007) and Haji-Khamneh & Harris (2009) that reported a dominant contribution of polarity cues for determining the PU. This could be due to the different everyday indoor scenes presented in both studies. Mittelstaedt (1986) reported large variation in the contribution of polarity to rod adjustment across participants and even across trials, suggesting the unstable influence of polarity on the SVV. This inconsistency might have arisen because of the difference in strategies in estimating the SVV and PU (Dyde et al., 2006). In the Oriented Character Recognition Test (OCHART) that measures PU (Dyde et al., 2006), an ambiguous symbol “p” is presented at a random orientation, and the participant judges whether it appears as a “p” or “d.” This task requires polarity judgment of the target, in which participants might actively refer to the polarity cues in the scene to identify the polarized symbol. On the other hand, non-polarized rod adjustment might require fewer reference demands than polarized objects. Rather, in the SVV task, a more straightforward strategy may be to adjust the rod to the straight lines in the scene, which might explain the largest contribution of frame cues in the rod adjustment task. As Dyde et al. (2006) speculated, the SVV might be closely linked to physical properties in spatial vision and may be involved in action control, whereas the PU is likely linked to object recognition.

Involvement of the SVV in action control as suggested by Dyde et al. (2006) might be limited because the relative contributions of visual cues to head tilt showed a different pattern from the SVV results in the present study. The relative contributions of visual cues to head tilt were not clearly different from each other, whereas those to the SVV showed distinct differences across visual cues. This dissociation could also be explained by the different demands of the tasks. Maintaining an upright posture requires muscle activation against gravity; therefore, information about upright direction derived from the polarity of an object should be important for postural control, whereas polarity is less informative for non-polarized rod adjustment. There is much evidence that primate spatial vision and action flexibly switch representation of coordinates including the following: retinotopic (Wandell, Dumoulin, & Brewer, 2007), eye-centered (Cohen & Andersen, 2002; Hallett & Lightstone, 1976), body-centered (Duhamel, Colby, & Goldberg, 1992; Kravitz, Saleem, Baker, & Mishkin, 2011; Sasaki, Anzai, Angelaki, & DeAngelis, 2020; Snyder, Grieve, Brotchie, & Andersen, 1998; Zhang, Heuer, & Britten, 2004), body segment centered (Duhamel, Colby, & Goldberg, 1998; Fogassi, Gallese, Fadiga, Luppino, Matelli, & Rizzolatti, 1996; Graziano & Gross, 1993), environmental (Kravitz et al., 2011; Sasaki et al., 2020; Snyder et al., 1998), and especially gravitational (Kheradmand & Winnick, 2017). Different contributions of visual cues across the SVV, PU, and postural control suggest that multiple representations of environmental coordinates could be used flexibly, possibly depending on task demand.

### Relationship between head tilt and SVV error

We did not find significant correlations between corresponding visual weights of head tilt and SVV error. This does not support our prediction that perceived body tilt induced by visual orientation (Cian et al., 1995; Ebenholtz & Benzschawel, 1977; Sigman et al., 1979) would produce a compensating postural response. However, lack of correlations could be attributed to different body postures, a seated position in the rod adjustment task, and a standing position in the head measurement task. Given that ankle joints are most sensitive to body inclination (Fitzpatrick & McCloskey, 1994) and, based on sensory reweighting theory (Nashner & Berthoz, 1978; Peterka, 2002), larger visual weights in the SVV task than the head tilt task could be due to increased proprioceptive inputs in the standing position. On the other hand, because the weight of proprioceptive inputs from the ankles varies considerably across individuals (Kluzik, Horak, & Peterka, 2005), the amount of proprioceptive influence could be different across participants, resulting in a lack of clear correlations between cue weights for head tilt and SVV.

### Upright preference and ocular torsion

We found a sharp accumulation of responses for deviation of scenes around the upright scene orientation. This is not surprising, as the effect of cues converges at around 0°, but it may also be related to an upright preference in scene perception. Recent studies using functional magnetic resonance imaging revealed that the visual system is most responsive to upright scenes represented in the scene-selective visual cortical area (Epstein, Higgins, Parker, Aguirre, & Cooperman, 2006; Nasr & Tootell, 2012). The visual system might be especially sensitive to deviation of scenes from the upright orientation, producing the greatest responses at around 0° scene orientation in head tilt and SVV error.

In addition, ocular torsion might have contributed to the current results, which is often produced as a compensatory eye rotation for body tilt (Dieterich & Brandt, 2019), but could also be produced visually by a static, tilted display (Goodenough, Sigman, Oltman, Rosso, & Mertz, 1979; Hughes, 1973; Pansell, Sverkersten, & Ygge, 2006; Sverkersten, Ygge, & Pansell, 2009). The torsional response might rotate retinal images and thus induce a shift in the perceived vertical, resulting in postural and rod adjustment error. However, with our current experimental setup, we cannot measure the extent of retinal torsion. Thus, the causal relationship between the visual torsion and postural/perceptual biases should be investigated in future research.

One may also associate the result of a sharp accumulation of responses around the upright orientation with ocular torsion, but it is not likely in this context because of the slightly different profiles of ocular torsion and the current results; visually induced torsion stays asymptotic at 45° of scene orientation (Pansell et al., 2006), whereas postural and perceptual biases seem to begin to disappear before 45°.

## Conclusions

We demonstrated substantial contributions of multiple visual cues to orientation for postural adjustment and the subjective visual vertical that were modulated as a function of scene tilt. This indicates that multiple visual processes are involved in postural control including orientation perception, surface perception and object/scene recognition. The relative contributions of these visual cues are not consistent between the two measures, suggesting different use of environmental coordinate representations for postural control and visual perception.
